# Limitations of recreational camera traps for wildlife management and conservation research: A practitioner’s perspective

**DOI:** 10.1007/s13280-015-0713-1

**Published:** 2015-10-27

**Authors:** Scott Newey, Paul Davidson, Sajid Nazir, Gorry Fairhurst, Fabio Verdicchio, R. Justin Irvine, René van der Wal

**Affiliations:** The James Hutton Institute, Craigiebuckler, Aberdeen, AB15 8QH UK; Faculty of Applied Ecology, Hedmark University College, Evenstad, 2480 Koppang, Norway; School of Biological Sciences, University of Aberdeen, Aberdeen, AB24 3UU UK; dot.rural, University of Aberdeen, Aberdeen, AB24 5UA UK; Electronics Research Group, School of Engineering, University of Aberdeen, Aberdeen, AB24 3UE UK; Aberdeen Centre for Environmental Sustainability, School of Biological Sciences, University of Aberdeen, Aberdeen, AB24 3UU UK

**Keywords:** Camera trap, Digital innovation, False negative, False positive, Sensors, Trail camera

## Abstract

The availability of affordable ‘recreational’ camera traps has dramatically increased over the last decade. We present survey results which show that many conservation practitioners use cheaper ‘recreational’ units for research rather than more expensive ‘professional’ equipment. We present our perspective of using two popular models of ‘recreational’ camera trap for ecological field-based studies. The models used (for >2 years) presented us with a range of practical problems at all stages of their use including deployment, operation, and data management, which collectively crippled data collection and limited opportunities for quantification of key issues arising. Our experiences demonstrate that prospective users need to have a sufficient understanding of the limitations camera trap technology poses, dimensions we communicate here. While the merits of different camera traps will be study specific, the performance of more expensive ‘professional’ models may prove more cost-effective in the long-term when using camera traps for research.

## Introduction

Camera trapping, the use of remotely triggered cameras that automatically take images of animals passing in front of the camera, is hugely popular with wildlife enthusiasts and recreational hunters who want to detect the presence of animals of interest. As with many other digital technologies, camera traps (also known as trail cameras) are now relatively low cost and easy-to-use. As a result, wildlife managers and conservationists are increasingly making use of this equipment for surveying and monitoring wild animals (Rowcliffe and Carbone 2008; O’Connell et al. 2011; McCallum 2013).

Effective wildlife management and conservation require reliable monitoring data. Conventional wildlife monitoring generally relies on resource intensive fieldwork. With increasing need for data, and generally decreasing resources for monitoring, one of the challenges for natural resource management is to develop more cost-effective approaches to ecological monitoring while ensuring that the data are robust and fit for purpose (Yoccoz et al. 2001; Legg and Nagy 2006). The deployment of camera traps is therefore an attractive tool because of their potential to provide a low cost, non-invasive survey method which (due to the physical absence of an observer) reduces disturbance and does not require the capture and handling of the study animals. Modern units, once set up and deployed, can be left unattended for days, weeks or even months with the potential to gather large amounts of data, thus overcoming some of the financial and logistical demands of monitoring effectively (Silveira et al. 2003). These characteristics make camera traps particularly well suited to the monitoring of elusive species in remote areas or to situations where population densities are so low that data gathering in more conventional ways would not be effective (Long et al. 2008).

Perhaps not surprisingly, therefore, and fuelled at least by their apparent simplicity, the use of camera traps in wildlife management and conservation research has grown enormously over the last 20 years, with camera traps being deployed in a huge range of projects (Rowcliffe and Carbone 2008; McCallum 2013; Verma et al. 2015). However, camera traps are not without problems; the often large amounts of data generated by these devices can overwhelm users and lead to problems with storage, backup, sharing and image processing (Harris et al. 2010; Sundaresan et al. 2011; Hamel et al. 2013). Moreover, recent literature reviews have highlighted issues around the design and utility of camera traps relating to their ability to produce the rigorous, unbiased and ecologically meaningful data that ecologists and other users expect (Meek and Pittet 2012; Burton et al. 2015; Meek et al. 2015a).

There are many makes and models of camera trap on the market. These range from expensive units designed for professional research use that offer a wide choice of settings and functions, high reliability, and some basic image management tools, to cheaper models with limited functions and less rigorous performance designed for recreational and amateur use^1^ (Swann et al. 2011; Meek et al. 2012). Differences in functions and reliability, however, also come with a difference in price, with ‘professional’ camera traps (e.g. Reconyx Hyperfire PC800) costing around three times as much as mid-range units (e.g. Bushnell TrophyCam HD 119737). The additional cost of ‘professional’ or high-end cameras compared to cheaper ‘recreational’ models, combined with limited budgets, the need to purchase a sufficient number of cameras and the apparent similarity in their specifications, mean that researchers and managers often choose to buy less expensive units to reduce costs and maximise replication and/or spatial coverage on the assumption that cheaper models will perform adequately (Rovero et al. 2013; Meek et al. 2015a). Notwithstanding the recent reviews of issues that camera trap users have experienced (Meek et al. 2015a), it is likely that problems experienced in camera trap studies may not be publicised because the main reason for using this technology is to report on ecological insights rather that technical difficulties. Moreover, where technical difficulties lead to data of insufficient ecological value the study, and therefore any issues with the equipment, is unlikely to emerge in the scientific literature.

To help fill this void, we report on two case studies which highlight some of the wide-ranging practical and operational issues practitioners should be aware of, and present information on the magnitude of some of these issues. Before doing so, we first present the results from a brief survey of the makes of camera trap used for research by UK government and non-government organisations and their motivations, along with a breakdown of the type of camera traps used in the peer-reviewed literature published in 2014.

## Survey of camera traps used by UK (non-) government agencies and the international research community

To find out what types of camera trap are used for wildlife and conservation research by practitioners, and on what grounds choices for certain makes and models are made, we contacted the UK’s major environmental governmental (GO) and non-governmental (NGO) organisations by email to extract those insights based on a short number of questions. To ensure that our sample included the UK’s most frequent users of camera traps, we asked our informants to also recommend other regular users of this technology for research and contacted those (thus employing snowball sampling). In addition we identified the model(s) of camera trap used from the methods sections (or by contacting the authors directly) of papers published in 2014 found using a Web of Science search on the term ‘camera trap*’^2^ (data retrieved 09/02/2015). Make and model of camera trap were assigned to three quality categories based on Meek et al. (2012), namely low-, middle- and high-end camera traps (Table 1). Where a particular model used was not listed we obtained the current cost from www.trailcampro.com or the manufacturer’s website (data retrieved 09/02/2015). This allowed us to compare the camera makes and models used in (non-)government agencies and charities with those used in the more academically orientated research conducted by higher education and research institutes.

**Table 1:** Type of camera, defined by approximate cost, used by five UK governmental and non-governmental organisations carrying out wildlife research, monitoring or surveys using camera traps that responded to requests for information. Circle size represents qualitative frequency of use: ‘frequently’—large circle, ‘occasionally’—middle size circle, and ‘rarely’—small circle. Regarding peer-reviewed literature (final column), only studies that actually used camera traps for wildlife research or monitoring and for which we were able to obtain a copy were included. Camera trap quality follows classification of Meek and Pittet (2012), cost categories in US Dollars are approximate

Camera trap quality	UK NGOs and governmental organisations					Peer-reviewed literature
	1	2	3	4	5	
Low-end (<300 USD)						(38 %, *n* = 10)
Mid-range (301–370 USD)						(23 %, *n* = 6)
High-end (371–740 USD)		–		–	–	(38 %, *n* = 10)

We found that UK government and non-government organisations predominantly used low-end and mid-range camera trap models (Table 1); only one of the six organisations for which information was obtained used primarily high-end models. Cost was often provided as the main reason for purchasing a particular make and model of camera trap, for example: “*We tend to use Reconyx for our major projects due to speed of image but Bushnell a lot on reserves as they are cheaper*” [organisation A]. Despite being aware of some of the advantages high-end models bring over cheaper models respondents indicated that lower-end cameras were purchased simply because there was not the resources to purchase the required number of high-end cameras: “*Others have recently recommended the Reconyx as being the most sensitive cameras, with less false negatives, but obviously there are cost implications of replacing all existing cameras with another that also might soon be outdated!*” [organisation D]; or “*… we needed quite a few cameras, so they also had to be affordable. Very few cameras met the spec, I don’t think Reconyx had anything suitable (or if they did, their price was very high)*” [organisation E]. This appeared particularly important where a purchase concerned a larger number of cameras: “*…we have a limited budget for work and provided we can get a device to do what is required I see no need to spend more. I think some companies such as Spypoint and Reconyx probably do have better quality control, and better supporting information giving an overall slightly better made, slicker and more user-friendly product, but when purchasing in bulk for a project the price differential means that provided the cheaper option works then you can gather twice as much data for the same money*” [organisation E]. Thus, when deciding on what camera traps to purchase, users were clearly willing to trade ‘quality’ for ‘quantity’ thereby hoping that the cheaper models would bring similar benefits to the substantially more expensive ones earmarked for research. Several respondents remarked that off-the-shelf camera traps lack flexibility or ability to upgrade. The former led one organisation to use bespoke units for certain projects where specific features were required. Yet, another organisation reported to sometimes make their own camera traps in order to meet specific requirements while undercutting the high costs of off-the-shelf or bespoke solutions: “*In the end I went to Maplin, got some off-the-shelf CCTV Kit (with a digital recorder) for a few £100s, then bunged it all in a big Peli Case running off leisure batteries and it worked fine; total cost was a fraction of the bespoke system [name removed] recommended*” [organisation F].

In the peer-reviewed literature published in 2014, more than 50 % of studies employed mid-range and low-end camera trap models though more than a third used high-end models (Table 1). The reasons for camera choice are not given in the literature, but our investigation clearly shows that non-professional camera traps are widely used for research.

## Case studies

We employed camera traps as part of an ecological study to investigate the occurrence of wild animals around deer carcasses in a remote and exposed location in the Scottish mountains during winter (Case Study 1). Despite being aware, in general terms, of key camera trap deployment issues, we opted to use this technology and purchased Bushnell TrophyCam camera traps, which are widely available mid-range units commonly used by recreational hunters and naturalists, but which have also been used for ecological research (e.g. Somaweera et al. 2011). Choosing these units allowed us to achieve the desired level of replication and spatial coverage within our budget. Subsequently, because of the issues we encountered with using our camera traps (see below; Case Study 1), we attempted to assess the performance of these devices to reliably detect and record the true presence of animals, in terms of both false positives (when the camera records an image when there is no animal present) and false negatives (when the camera fails to record the presence of an animal) (Case Study 2).

In presenting these case studies we seek to illustrate some of our experiences of deploying a widely used ‘recreational’ camera trap in ecological research. Our aim is not to critique a particular make or model or compare it with different makes and models of camera traps (such studies already exist; see, for example, Hughson et al. 2010; Weingarth et al. 2013). Instead our aim is to raise awareness of the wide range of practical issues that may be experienced when using camera traps for research.

### Case Study 1—Monitoring wildlife activity around deer carcasses: Researcher experiences

**Fig. 1:**
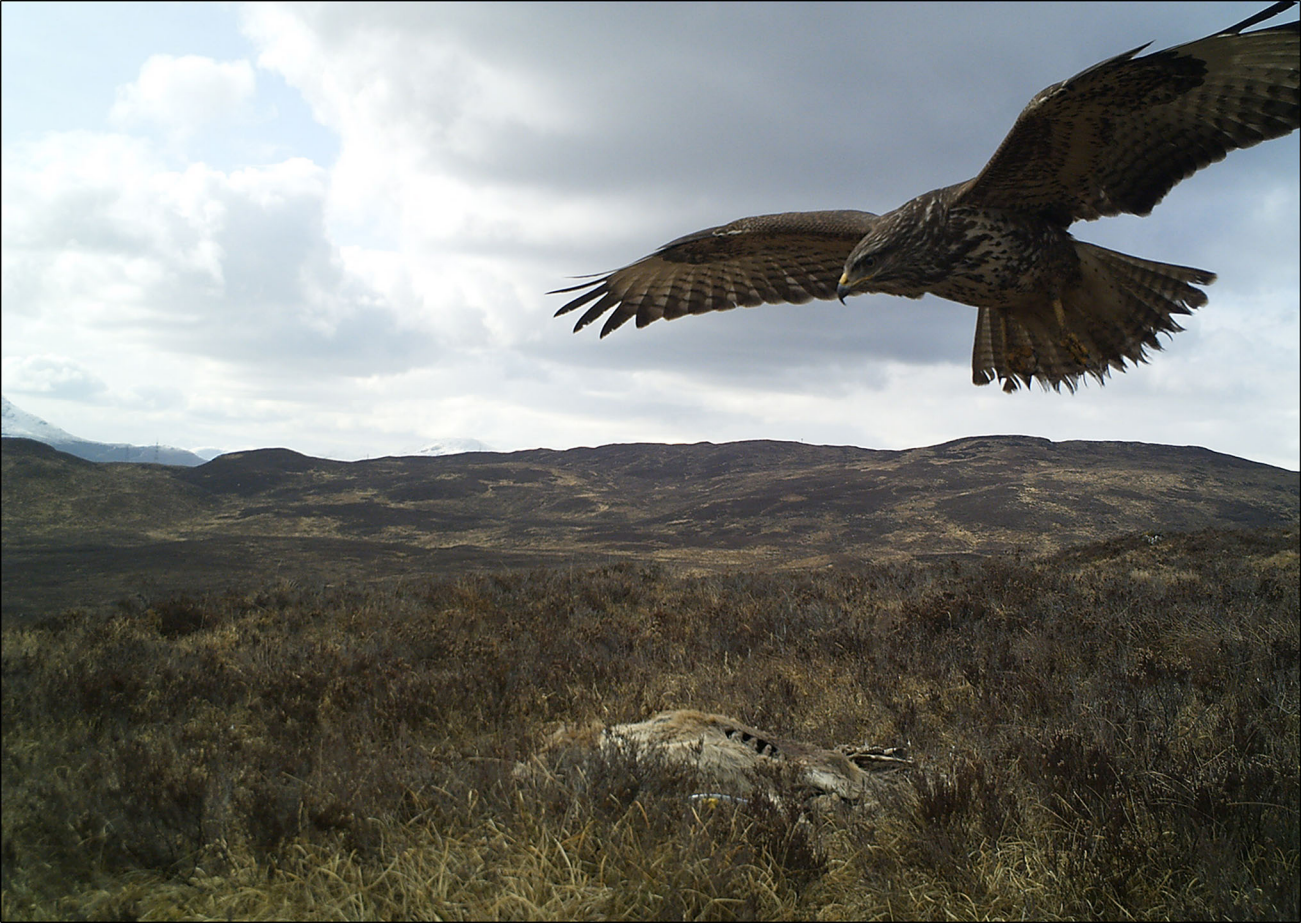
Image of a buzzard (*Buteo buteo*) at a deer carcass captured by one of our camera traps

Camera traps (Bushnell, Trophy Cam, model 119435) were set up at 4 m distance from fresh red deer (*Cervus elaphus*) carcasses on heather (*Calluna vulgaris*) dominated moorland (550 m above sea level) on a private shooting estate in the Cairngorms National Park with the aim of detecting visiting scavengers. This research was conducted to further the debate on carcass placement as a nature conservation tool (Fig. 1, see Fielding et al. 2013). Carcasses were placed out in winter during the deer shooting season and we initially monitored three carcass sites. Camera traps were set to ‘normal’ sensitivity, and attached to wooden posts so that the passive infrared (PIR) sensor was at 0.6 m above ground level. The camera was programmed to take three images per trigger event with a 1 s delay before the camera was armed again. Routinely only one camera (denoted ‘primary camera’; generally facing north-west) was placed at each carcass site, but additional units (denoted ‘confirmatory camera’) were rotated around the carcass sites where they were set up in the same way, but at 90° to the primary camera (and generally facing north-east). All cameras were set up so that the carcass was in the centre of each camera’s field of view. Carcasses and cameras were placed out in November 2011 and monitored for 2 years with batteries and memory cards changed every 2–6 weeks depending on weather, which during periods of heavy snow fall and ice limited access to the study sites. In addition, there was a camera placed at each of two (carcass-free) control sites.

#### Deployment issues

**Fig. 2:**
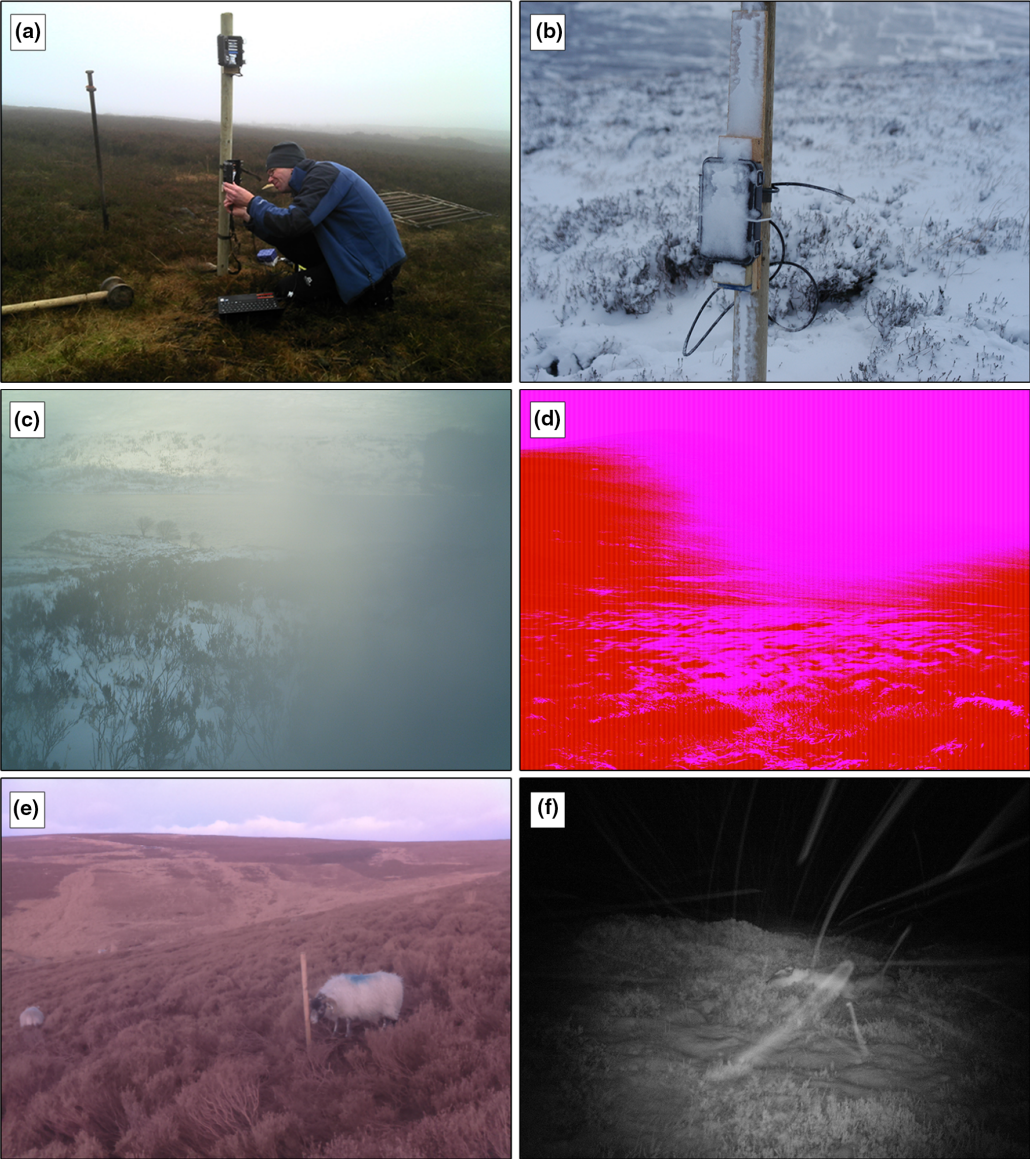
A collection of images showing some of the key aspects of camera trap use in our study: **a** setting up cameras—here using a laptop to download and view images to test camera alignment (walk-by test) potentially exposing insides of the camera trap, SD card, and computer or other viewing device, to the elements; **b** sensor and camera blocked by snow leading to no photos being taken; **c** image obscured by sleet gathering in the aperture; **d** an unusual (but not rare) malfunction of the camera; **e** sheep caught by time-lapse camera trap but *not* the motion-activated camera trap; **f** wind and snow activity triggering a false positive during the night

Camera setup, via the pre-programmed menus, was technically straightforward but practically rather fiddly because the buttons were small (especially problematic when having cold fingers or wearing gloves), and the screen was difficult to read in low light or bright sunlight. To enable comparison between images from multiple cameras, it was important to synchronise the camera’s internal clocks and to be able to identify which camera images came from. However, synchronising time across multiple cameras turned out to be time consuming and imprecise, and there was no functionality to record camera or site details either on the image or as meta-data within an image header file. In an attempt to overcome this, all cameras were set up in the laboratory prior to deployment. Despite this preparation, we found that in many cases cameras lost their settings by the time we came to deploy the units on site—presumably because, during transit, battery power was temporarily lost due to bumps and vibrations while travelling to the site by off-road vehicle. Therefore, it became necessary to reconfigure the camera settings and synchronise camera clocks in the field often under wet, windy or snowy conditions. Once a camera setup was completed, a ‘walk-by test’ was carried out to determine if the camera was triggered by movement at the target location. However, this also proved awkward because the camera traps used had no in-built image viewing screen and either required the image to be downloaded from the camera onto a laptop or the SD card to be inserted into a digital camera in order to view it, with obvious implications in poor weather (Fig. 2a).

#### Operational issues

Although camera traps boast long battery life and can collect and store tens of thousands of images unattended, they still require regular visits to retrieve data, change batteries and ensure the camera is functioning correctly (e.g. still aimed at the target area). Initially, we powered camera traps using eight AA cell alkali, and later lithium, batteries which were supposed to provide up to 12 months power. However, we found that in winter, when cameras were using a lot of flash and recorded a large number of images (10 000 or more per day; data not shown), batteries expired within around 3 days, and even a moderate number of images of around 10 000 per month exhausted a set of batteries in 4–6 weeks, resulting in many days with no data collection. Moreover, and critically, we observed that the camera’s internal clock would commonly reset itself to the default factory setting multiple times between visits, possibly due to changes in battery voltage with ambient temperature or temporarily lost battery connection (potentially due to the wind shaking the camera or mounting post). This essentially rendered the majority of images unusable as it was not possible to identify the actual date and time that a photograph was recorded. Substantial data loss was also caused by unsolicited changes to other camera settings, presumably also associated with a camera re-setting itself due to changes in, or loss of, battery power. There was further loss of data due to obstruction of the lens by condensation, and snow or ice build-up on the camera (Fig. 2b).

#### Data management issues

Clock re-setting rendered it impossible to compare corresponding time periods among the carcasses. This aside, cameras typically captured around 2000–10 000 images per month of deployment which, with six cameras, yielded 20 000–60 000 images per month. We rapidly fell behind in cataloguing images, a problem exacerbated by the lack of in-built tools to facilitate image and data management (e.g. recording and access to image meta-data) and by problems sharing such a large number of images with project partners based in other institutions.

**Fig. 3:**
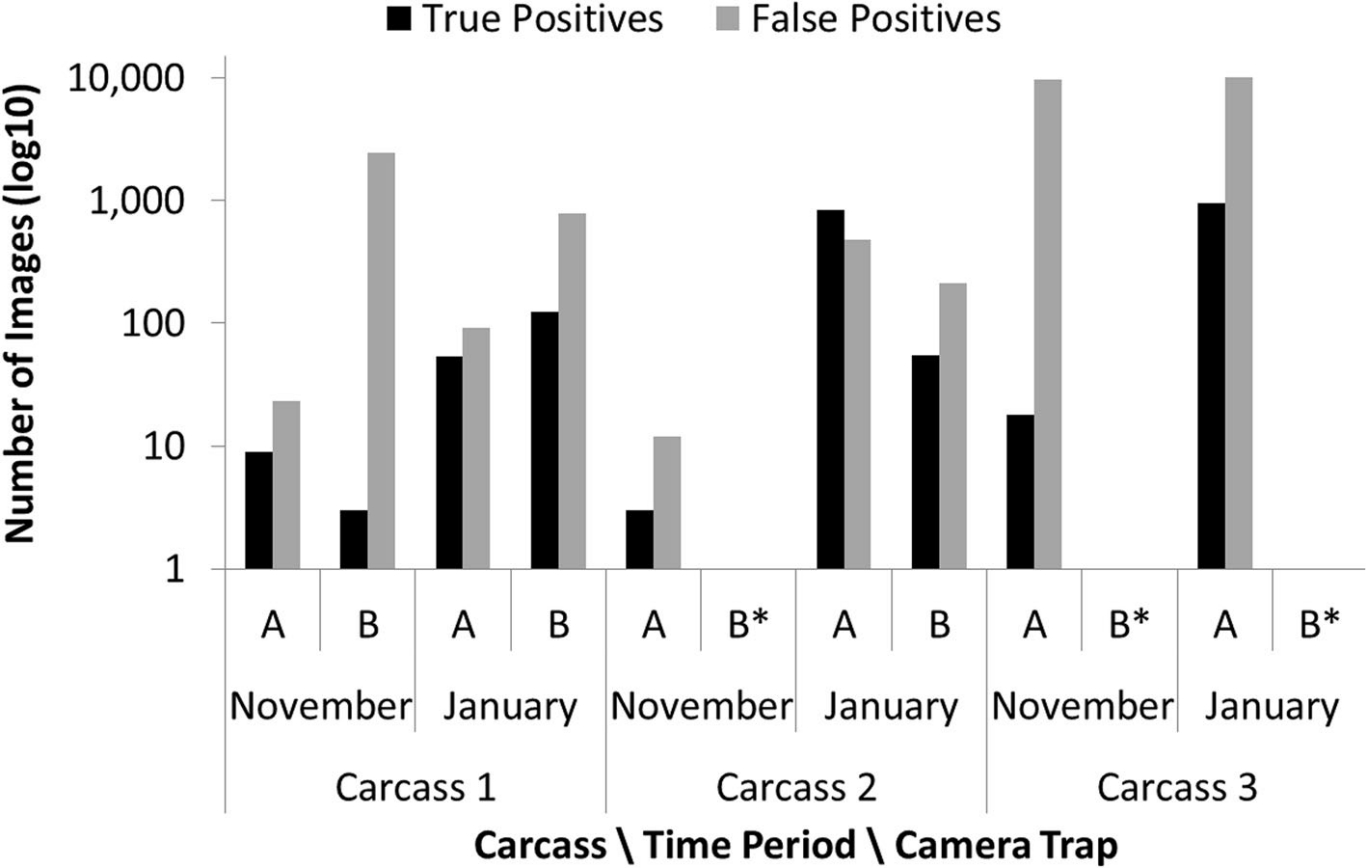
The number of images and number of false positive images recorded during nine camera trap deployments (Camera ‘A’—primary camera, ‘B’—confirmatory camera) at three deer carcass sites on two sampling periods (November 2011, January 2012). The height of each *bar* shows the number of images of each category captured during each deployment. The numbers of images are shown on the Log_10_ scale to account for the large differences between sites. Cameras *A* and *B* were of the same type and monitoring the same target area at the same time and thus would be expected to record a similar number of images. ‘*’ indicates that no confirmatory camera was deployed

Peculiarly, we found a very high proportion, and therefore large numbers, of false positive images (Fig. 3). False positives imposed a substantial drain on resources, in terms of battery power, on-board storage capacity, network storage capacity and time needed for image processing and data extraction.

To illustrate some of the issues we present data from two periods of 2 weeks each, in November 2011 and January 2012; these were the only periods when time settings did not change, allowing us to carry out a comparative analysis. During these two periods, over 25 000 images were collected (we report on the number of images rather than animal visits because the problems with clocks re-setting and asynchrony prevented us from identifying distinct episodes of animal activity). Both the total number of images (15–10 965 per camera per deployment) and the percentage of false positive images (36–99 %) differed greatly among carcass sites, as well as between cameras within the same carcass site (Fig. 3). Disturbingly, on the three occasions when two cameras were monitoring the same carcass there were large differences in the number of recorded images between the two cameras, demonstrating that cameras were failing to detect some wildlife activity at a carcass (Fig. 3). For example, at site 1 in November the primary camera recorded 32 images of which 9 were true positives (contained images of animals), compared to the confirmatory camera which recorded 2459 images of which only 3 were true positive images (Fig. 3). Therefore, the confirmatory camera failed to record (i.e. false negatives) six images of wildlife activity relative to the primary camera, while accruing a far greater number of false positives. We tested whether the high proportion of false positives was due to faulty cameras by running all the cameras used inside dark boxes, but no images were recorded indicating that there must be one or more other causes for the high proportion of false positives experienced in the field. Despite these issues, the data revealed the presence of raven (*Corvus corax*), red fox (*Vulpes vulpes*), mountain hare (*Lepus timidus*), and red deer (*Cervus elaphus*) at the carcass sites, but only one of the seven cameras deployed detected all animal species known to have visited a particular carcass. In summary, our expectations for collecting quantitative data on the occurrence of scavengers over time from carcasses in remote locations were frustrated by the combination of deployment, operational and data management issues. In particular, the loss of time settings and the large number of false positive imagery were problematic, resulting in very limited data (Table 2). That we also found differences between camera traps monitoring the same carcass, and evidence that camera traps were sometimes failing to detect animals at a carcass, raised questions of the reliability of these camera traps for our purposes under these conditions.

**Table 2:** Summary of practical issues encountered while using recreational camera traps for research in two case studies and their effect on our experimental process and outcomes

Problems during use	Consequences	Case study
Deployment		
Fiddly navigation—small buttons	Increasing time needed for fieldwork and increased error rate	1, 2
Screen is difficult to read in low light or bright sunlight	Increasing time needed for fieldwork and increased error rate	1, 2
Synchronising time consuming and awkward	Increased setup and deployment and approximate synchronisation	1, 2
Keeping track of cameras and images (no meta-data)	Increased post-collection processing time, risk of introducing errors and data loss	1, 2
Losing settings during transit	Increasing time needed for fieldwork, error rate, and loss of data if not detected and corrected	1
Walk-by test requires downloading image on laptop in the field	Time consuming and requires access to laptop in the field	1, 2
Operational		
Excessive use of flash and frequent triggering (mostly generating false positives)	Swift depletion of batteries; requiring additional field visits to replace batteries; increased costs	
Internal clocks would commonly reset to factory settings	Loss of useable data or loss of data quality	1
Snow/sleet and ice build-up and condensation on lens	Poor quality or no usable imagery	1, 2
Camera failure due to unknown causes	Loss of data	1, 2
Loss of clock synchrony between cameras, with rate of divergence changing over deployment period	Loss of useable data or loss of data quality	1, 2
Data management		
Loss of meaningful date-time stamps	Rendered large volumes of data useless (and sampling effort could not be assessed)	1, 2
Large number of images	Problems sharing data. Difficulties cataloguing and analysing	1, 2
High proportion of false positives	Drains battery power, on-board storage, network storage, time for processing, data extraction	1
Differences in the number of animal detections among cameras monitoring the same carcass	Missed data due to questionable effectiveness of camera traps	1, 2
Highly variable proportion of false positives/negatives between locations, time periods and cameras	Questioning camera traps as a research tool. Potential biases, systematic difference between cameras	1, 2
Lack of tools to either simultaneously log or match external data sources to imagery	Labour-intensive to extract and match images from multiple cameras with meteorological data	2

### Case Study 2—Determining the causes of false positives: Trials with sheep

Intrigued by the high proportions of false positives recorded in Case Study 1, we carried out trials to determine their causes. We suspected that weather conditions (in particular wind) and vegetation height (especially tall swards) might be causing localised changes in temperature within the PIR’s detection zone, triggering the camera without an animal being present (Meek et al. 2012). We speculated that the height of the camera (more specifically the PIR), above the ground or above the top of moving vegetation might also influence the occurrence of false positives. To simplify logistics, trials were carried out during March–April 2014 at the James Hutton Institute’s upland research farm in Aberdeenshire (56.8959°N, 2.5445°W) in an area of moorland (at 350 m above sea level) comprising a heather–grass mosaic used for sheep grazing. Using this site also allowed us to make use of weather data collected at an Environmental Change Network^3^ meteorological station at this location.

We set up motion-activated and time-lapse camera traps trained on a sheep feed block placed at 4 m distance from the cameras. The time-lapse camera served as a reference camera providing ‘true’ reference images against which to compare images from a motion-activated (Bushnell) camera. To investigate the effects of vegetation height on camera performance, the experiment was carried out at two sites about 200 m apart; a tall vegetation site with a heather sward of 30–50 cm height and a short vegetation site of heavily grazed grass. To assess the effects of camera height on camera performance we deployed two motion-activated camera traps (Bushnell TrophyCam, model 119536) on the same post at 1.2 and 0.6 m above the ground (Fig. 2a). These camera traps were programmed to record a single image per motion-activated trigger event, with a 5-s delay between potential triggers, and with the PIR sensitivity set to ‘automatic.’ To determine whether motion-activated cameras were failing to detect activity around feed blocks an additional prototype camera (WiseEye—Nazir et al. 2014) was mounted, immediately next to the Bushnell camera traps, at 1.2 m above the ground and recording a time-lapse image every 2 min. Cameras were run for 2–4 days at a time at each site, allowing them to encounter variation in weather conditions.

To assess the response of camera traps in relation to the presence or absence of animals (recorded by the time-lapse, reference, camera trap), we viewed and compared each 2-min time-lapse image against the corresponding record obtained from each motion-activated camera trap at the same time. The response of the motion-activated camera traps compared to the corresponding time-lapse image (±20 s) was classified as follows: true positive when the image from the time-lapse camera and the motion-activated camera images both showed a sheep; true negative when the time-lapse image did not contain a sheep and the motion-activated camera trap had not recorded an image; false positive when the time-lapse image did not show a sheep but the motion-activated camera had recorded an image at the same time; or false negative when the time-lapse image included sheep, but the motion-activated camera had not recorded an image.

#### Deployment issues

It was critical that the internal clocks of the motion-activated cameras and the time-lapse camera remained precisely synchronised in order to assess the occurrence of true and false detections. All cameras were therefore synchronised to within ±3 s which, along with the rest of process of setting up multiple cameras, was time consuming because all (but the reference) camera setup had to be done manually (Fig. 2a).

#### Operational issues

Camera failure, due to unknown causes and re-setting of the camera traps’ time settings, meant that data from some trials had to be discarded, in addition to that lost due to precipitation obscuring the lens—although this was less of an issue than in Case Study 1 (Fig. 2c, d). However, the most significant operational issue was due to loss of synchrony in time between units, and peculiarly that the degree and rate of divergence changed over each 2–4 day trial period.

#### Data management issues

Processing the large amount of data collected by multiple camera traps we had deployed was greatly hampered by the problem of changing asynchrony between the camera trap units. This not only made comparing images from different cameras difficult and very time consuming, if not impossible in many cases, but also obstructed efforts to link imagery to corresponding (time-stamped) meteorological data. This meant that relevant weather variables had to be manually extracted from the meteorological data, matched and linked with the appropriate imagery and then augmented by visual assessment of weather from images, a process that took us approximately 14 h to complete for every 1000 images.

Due to the challenges described above we were only able to analyse a small subset of camera trap data (three 6-h long deployment periods—see Table 3). To our surprise, no false positives were detected during those short observation periods. What we did find, however, was that the motion-activated camera failed to detect (i.e. false negatives) 49–68 % of the sheep shown to be present by the time-lapse data (Table 3; Fig. 2e). That we were only able to analyse a subset of the camera trap data, along with the fact that meteorological variables were recorded hourly, meant that the camera trap data spanned too short a time interval for us to investigate potential effects of weather, vegetation height, or camera height on the rather alarming numbers of false negative images generated. In summary, we had envisaged collecting a large quantity of high-quality, time-stamped data from a well-replicated field trial. Instead, due to the practical set-backs, we ended up having to use a much smaller number of images while using a disproportionate amount of time to sort and manually link imagery to meteorological and visual weather data (Table 2).

**Table 3:** Summary findings from Case Study 2 comparing time-lapse images with corresponding records from a motion-activated camera trap deployed at two different heights (see Fig. 1a) in either tall heather of short grass sward. ‘No. sheep visits’ is the (real) number of sheep visits recorded by the time-lapse camera against which each motion-activated camera was compared. ‘False negative records’ are the number of sheep visitations not detected by the motion-activated camera trap

Vegetation	Camera height (m)	Time-lapse camera		Motion-activated camera	
		Total no. images^a^	No. sheep visits	No. sheep visits detected	False negative records (%)^b^
Short grass	1.2	181	95	30	68
Tall heather	0.6	181	71	27	62
Tall heather	1.2	181	71	36	49

^a^The time-lapse camera recorded one image every 2 min for 6 h giving a total of 181 time-lapse images

^b^Percentage based on the number of sheep visits recorded by the time-lapse camera

## Discussion

Our choice of camera trap was based on balancing available funding, cost per unit and the number of sites we wished to monitor. Our exploration of camera trap use among UK governmental and non-governmental organisations, and also the peer-reviewed literature, suggests that such compromise-based decisions on the choice of camera trap model are widespread. Among the UK (non-) governmental organisations that provided us with information, cost was cited as the main reason for purchasing a particular camera trap.

Like many users, we were eager to use camera traps as a tool for monitoring elusive species that generally occur at low population density in remote locations. At the outset of our study we had a general awareness of some of the limitations reported in the literature. We believed that the potential benefits of using camera traps in conjunction with appropriate analytical methods would overcome the known challenges. However, our enthusiasm to use camera traps was quickly tempered by a range of problems such as large numbers of false positive imagery, cameras re-setting themselves, difficulties over meta-data which reduced the amount of useable data retrieved to quite low levels, and the huge effort required to extract the remaining useful data (see Table 2 for a catalogue of the problems encountered).

We recognise that our experiences will relate directly to the cameras we used and the environments we worked in, and may thus not be representative of other contexts. However, recent reviews suggest that low to middle range ‘recreational’ camera traps may have common performance issues, and that there is a growing trend towards greater use of ‘professional’ camera traps concurrent with increasing awareness of the some of the limitations often associated with ‘recreational’ models (Meek et al. 2015a, b).

While we acknowledge that our case studies are context specific, the experiences we report on here may help guide those not directly involved in camera trap research and who may have high expectations of the technology, but may be less aware of the potential advantages of deploying more expensive and reliable models (Meek et al. 2015a). The following sections provide some specific insights into the occurrence of false positives and negatives, two of the more widely acknowledged problems with camera traps, and our attempts to understand the causes of these.

### The issue of numerous false positives

Our experience shows that camera traps can generate large numbers of spurious detections (false positives) which can rapidly fill up memory cards, drain batteries and overwhelm available image storage capacity. Moreover, the subsequent need to process large numbers of images is very time consuming and delays, if not prevents, interpretation of data and its use in wildlife management and conservation. Our first case study brought out a huge variation in the number of false positives between cameras of the same make and model and between sites. The most exposed site of the three had by far the most false positives, which made us suspect that strong winds triggered images because the camera was detecting changes in temperature due to either moving vegetation (Fig. 2f) or movement of the camera and/or the post it was mounted on (either of which could have the same effect). Ironically, when setting up a field study to explicitly investigate key causes of false positives, a multitude of deployment, operational and data management issues prevented us from making progress (Table 2).

### The issue of false negatives

By their nature it is difficult to identify and quantify false negatives. However, our comparison with cameras recording regular time-lapse (Case Study 2) revealed a surprisingly high proportion of false negative responses. They present a serious issue as this may lead to animals being missed or species being under-represented in a study, leading to bias in subsequent analysis. Our results indicate that the camera traps used here failed to detect up to 68 % of verified animal activity with appreciable variation between individual cameras and deployments. We were once more unable to identify what factors were driving the high occurrence of false negatives because our attempts to do so were hampered by camera failure, problems synchronising images from different cameras within and between deployments, and difficulties matching imagery with meteorological data.

### PIR sensor, heat differential and stealth sheep

The detection technology on most camera traps is based on PIR sensors which monitor a volume of space for differences in temperature between an object in the PIR sensor’s detection zone and background levels (temperature differential) and motion, both of which must be present at the same time for an event to trigger the camera. Optimum conditions for PIR sensors require a temperature difference greater than 5 °C; thus the ability of a PIR sensor to detect movement is poor if ambient and external body temperatures are within 5 °C of each other (Meek et al. 2015a). If there is a target in front of the camera but there is insufficient difference or change in temperature, then the PIR may not detect the target and thus lead to a ‘false positive’. However, a change in temperature due to non-animal related events, such as warm air moving within the sensors’ range or moving vegetation that causes a thermal shadow, can trigger a PIR resulting in ‘false positive’ images. The insulating properties of an animal’s skin, fur or feathers, water particles in the air, or interplay between such factors, may mask an animal’s ‘heat signal’ and reduce the effectiveness of PIR sensors to detect animal presence, potentially leading to false negatives. For example, although the reasons why so many sheep were missed in Case Study 2 remain unclear, snow and water droplets that were observed to collect on their fleeces may have reduced the PIR’s sensors detection ability. Thus, there seem to be conditions under which PIR sensors may not be particularly effective at reliably triggering camera traps. This may be exasperated because many recreational camera traps tend to be optimised to detect the larger mammals and birds of interest to American and northern European hunters (Meek and Pittet 2012). Differences in camera sensitivity and in the detectability of individuals and species are well known (Nichols et al. 2011; Hamel et al. 2013; Rovero et al. 2013; Weingarth et al. 2013), and studies comparing different camera traps side-by-side show that there is considerable variation in the effectiveness of different makes and models in detecting the same species (Hughson et al. 2010; Weingarth et al. 2013). Nonetheless, that camera traps used here failed to detect a large proportion of sheep coming to a feed block only 4 m in front of camera traps, is a concern, and underlines the message that camera traps should be piloted prior to use in a particular study.

Given the variation in species detectability and the occurrence of false negatives Hamel et al. (2013) have suggested using time-lapse imagery rather than relying on motion-activated images because for the later the absence of an image cannot be unambiguously interpreted as no animal present. While time-lapse photography can still result in many images and associated challenges, it does allow for a robust interpretation of positive and negative images and provides a record of duration of camera functioning. Use of time-lapse, however, runs the risk of missing events that occur between time-lapse images, and in environments where animal density is low such an approach may not be appropriate (Hamel et al. 2013).

Good survey design and appropriate analytical methods can address some of the problems encountered with camera trap technology, and for example can accommodate differences in animal detectability (e.g. Royle et al. 2009; Gardner et al. 2010; O’Brien et al. 2010). However, these techniques still rely on robust data and the accompanying image meta-data, but our experiences demonstrate that securing these from camera trapping surveys may be more challenging than first appreciated. In addition, the capacity of camera traps to collect huge numbers of images, or video, and associated challenges for data management and processing pose a significant, but often under-rated challenge that users need to appreciate along with the deployment and operation issues raised here and in other recent reviews (Harris et al. 2010; Sundaresan et al. 2011; Burton et al. 2015; Meek et al. 2015a).

## Concluding remarks

Camera traps offer a powerful tool for studying and monitoring a range of wildlife, and their use is likely to continue to grow. Based on our experiences reported here, we urge practitioners to carefully consider the costs and benefits of different makes and models of camera trap. Some of the issues we report here may have been alleviated had we used ‘professional’ camera traps. Cheaper models may ostensibly offer similar features to high-end models for less capital outlay, but may lack the reliability and performance of more expensive ‘professional’ models that require a more substantial initial investment, but which may prove more cost-effective in the long-term. To assess the advantages and disadvantages of different camera traps it is essential that users have a sufficient understanding of the limitations associated with this technology and its applications in different settings. With potential problems in mind, some of which we highlight here, practitioners would be advised to carry out a pilot study comparing different camera traps to assess their suitability and identify problems, and find which model best meets their requirements.

We suggest three areas where camera trap manufacturers can contribute to developing user-friendly, flexible fit-for-purpose devices suitable for research. First, one of the most fundamental shortcomings of currently available commercial digital camera traps is that they are closed systems with limited options for customisation (Meek and Pittet 2012). Pre-defined user-selectable options mean that in principle most camera traps are simple to set up. However, they usually lack a user-friendly interface and the flexibility that would extend their utility by, for example, the option for users to add different detectors or other peripheral sensors to allow cameras traps to meet different research needs over their life-time. Second, image management would be improved if camera traps allowed images to contain a greater variety of, preferably user-definable, labels (e.g. site label) as standard meta-data tags which can be manipulated using common desktop software, as opposed to proprietary software. Third, while there are desktop applications for automating image processing (e.g. ‘ImageJ’,^4^ DISCOVERY^5^) the ability for camera traps to carry out on-board image processing in order to identify false positives, and either delete or mark them, would help with both storage space and post-field image management. While there are some very reliable high-end camera traps on the market suitable for a wide range of studies we believe there is a need for a flexible, modular, open-source camera trap platform that users can freely adapt to address specific research questions and exploit emerging technology, and which helps address some of the limitations associated with many commercially available camera traps.

## Footnotes

1 www.trailcampro.com.

2 We used the ‘*’ wildcard to capture all word endings for the ‘trap’ search term.

3 ECN, http://www.ecn.ac.uk/.

4 http://imagej.net.

5 http://www.biosch.hku.hk/ecology/staffhp/lk/Discovery/.
